# Metagenomic analysis of herbivorous mammalian viral communities in the Northwest Plateau

**DOI:** 10.1186/s12864-023-09646-1

**Published:** 2023-09-25

**Authors:** Jiamin Pan, Likai Ji, Haisheng Wu, Xiaochun Wang, Yan Wang, Yan Wu, Shixing Yang, Quan Shen, Yuwei Liu, Wen Zhang, Keshan Zhang, Tongling Shan

**Affiliations:** 1 Department of Laboratory Medicine, School of Medicine, Jiangsu University, Zhenjiang, Jiangsu China; 2 State Key Laboratory of Veterinary Etiological Biology, College of Veterinary Medicine, Lanzhou University, Lanzhou Veterinary Research Institute, Chinese Academy of Agricultural Sciences, Lanzhou, China; 3 Shanghai Veterinary Research Institute, Chinese Academy of Agricultural Sciences, Shanghai, 200241 China

**Keywords:** Viral metagenomics, Herbivorous mammalian, Northwestern plateau, Viral communities, Phylogenetic analysis

## Abstract

**Background:**

Mammals are potential hosts for many infectious diseases. However, studies on the viral communities of herbivorous mammals in the Northwest Plateau are limited. Here, we studied the viral communities of herbivorous mammals in the Northwest Plateau using virus metagenomic analysis to analyze and compare the viral community composition of seven animal species.

**Results:**

By library construction and next-generation sequencing, contigs and singlets reads with similar viral sequences were classified into 24 viral families. Analyzed from the perspective of sampling areas, the virus community composition was relatively similar in two areas of Wuwei and Jinchang, Gansu Province. Analyzed from the perspective of seven animal species, the viral reads of seven animal species were mostly ssDNA and dominated by CRESS-DNA viruses. Phylogenetic analysis based on viral marker genes indicated that CRESS-DNA viruses and microviruses have high genetic diversity. In addition to DNA viruses, nodaviruses, pepper mild mottle viruses and picornaviruses were RNA viruses that we performed by phylogenetic analysis. The CRESS-DNA viruses and nodaviruses are believed to infect plants and insects, and microviruses can infect bacteria, identifying that they were likely from the diet of herbivorous mammals. Notably, two picornaviruses were identified from red deer and wild horse, showing that the picornavirus found in red deer had the relatively high similarity with human hepatitis A virus, and the picornavirus carried by wild horse could potentially form a new species within the *Picornaviridae* family.

**Conclusions:**

This study explored the herbivorous mammalian virus community in the Northwest Plateau and the genetic characteristics of viruses that potentially threaten human health. It reveals the diversity and stability of herbivorous mammalian virus communities in the Northwest Plateau and helps to expand our knowledge of various herbivorous mammalian potentially pathogenic viruses.

**Supplementary Information:**

The online version contains supplementary material available at 10.1186/s12864-023-09646-1.

## Background

Herbivorous mammals play an important role in human survival, contributing to food, social as well as economic value. Optimizing the productivity, health, welfare and environmental footprint of herbivores, particularly ruminants, has been the subject of numerous global studies for decades. Many studies have focused on the gut of herbivorous mammals. In recent years, bacteria and methanogenic archaea have been the main focus of research efforts, in contrast to the under-analyzed viral communities of herbivorous mammals [1]. The herbivorous mammalian gut microbiota contains dense and diverse populations of viruses, most of which actively infect and replicate microbes residing in the gut, and viral genomes are often found integrated into the genomes of gut microbes. Viruses contribute to genetic transfer and cause microbial lysis, leading to the release of microbial enzymes and the regulation of microbial community diversity [1]. Additionally, herbivorous mammals are potential hosts for pathogenic viruses that pose a health risk to humans and other animals and are a particularly high risk source of zoonotic virus transmission [2].

Herbivorous mammals including domestic animals and wildlife are potential hosts of numerous mammalian infectious diseases. Rotaviruses, alpha-coronaviruses, and orthopoxviruses, which are widespread in herbivorous mammals, and coronaviruses undergo relatively frequent host jumps [3] in mammals. Domestic animals, especially cattle, sheep, goats and pigs, are susceptible to get foot and mouth disease [4]. In addition to domestic livestock, wild even-toed ungulates are also susceptible to get foot and mouth disease, and experimental infections have been reported in white-tailed deer (*Odocoileus virginianus*) [5, 6], mule deer (*Odocoileus hemionus*) [7], elk (*Cervus canadensis*), bison (*Bison bison*) [8] and fork-horned antelope (*Antilocapra americana*) [9] showed susceptibility. Although pigs are well known host animals for Hepatitis E Virus (HEV), relatively few studies have reported evidence of Hepatitis E Virus (HEV) infection in cattle, goat, camel, horse and deer [10].

Not only are domestic animals and wildlife potential hosts for mammals infectious disease, but many human infections also originate in herbivorous mammals, and thus the ongoing transmission of disease from animals to humans constitutes a major global health burden. This is particularly true where populations are dense and where the pressure on environmental and economic resources is greatest. It is estimated that more than one billion cases of zoonotic diseases occur each year, and emerging zoonoses have caused hundreds of billions of dollars in economic losses [2]. Given the rich diversity of animal species worldwide, it is not surprising that animals are the source of most human infectious diseases [11]. Some authors point out that emerging human diseases are often those that can be transmitted between animals and humans [12]. Zoonotic transmission of Rabies virus, camelpox virus, Middle East respiratory syndrome coronavirus and Crimean-Congo haemorrhagic fever virus [13] from camels has been reported.

The Northwest Plateau mainly includes the Loess Plateau, Qinghai-Tibet Plateau and Inner Mongolia Plateau, with an average altitude of over 4,000 m. Gansu Province is located at the intersection of the Loess Plateau, Qinghai-Tibet Plateau and Inner Mongolia Plateau, with long and narrow terrain, diverse landscapes and a dry climate, which is a continental temperate monsoon climate. There are more than 90 rare animal species under national protection, including 24 animal species belonging to the Class I protected animals, 24 animal species belonging to the Class II protected animals and 40 animal species belonging to the Class III protected animals [14]. At the same time, the Northwest Plateau is rich in grassland resources, with a large number and high diversity of herbivorous mammals. With global warming, the ecological environment of the plateau is becoming more and more fragile, and many animals are unable to adapt to the changes in the environment and are on the verge of extinction. Therefore, it is important to carry out research on herbivorous mammals of the plateau to maintain the ecological balance of the plateau.

In this study, we investigated the viral communities of seven animal species, including camel, cattle, donkey, goat, horse, red deer and yak, and analyzed the genetic diversity of the major viral communities of herbivorous mammals in the Northwest Plateau using a viral metagenomic approach.

## Materials and methods

### Sample collection and preparation

In December 2018, we collected a total of 298 samples from camel, cattle, donkey, goat, horse, red deer and yak in Wuwei and Jinchang, Gansu Province. Among them, 279 were fecal samples and 19 were oral swabs. We collected samples in farms and safari park and speciated the hosts of collected fecal samples by watching them defecate. The samples were grouped into 36 sample pools depending on the animal species and location of collection. All samples were collected by disposable material and shipped on dry ice. Fecal samples were taken non-invasively and were re-suspended in 10 volumes of phosphate-buffered saline (PBS) and vigorously vortexed for 5 min. Fecal supernatants were then collected after centrifugation (10 min, 15,000 × g). The tips of respiratory swabs were immersed into 1 ml PBS and vigorously vortexed for 5 min and incubated for 30 min at 4 °C. The supernatants were then collected after centrifugation (10 min, 15,000 × g).

### Viral metagenomic analysis

Five hundred microliters of each supernatant was filtered through a 0.45-μm filter (Millipore) to remove eukaryotic and bacterial cell-sized particles. The filtrate of concentrated viral particles was treated with DNase and RNase to digest unprotected nucleic acids at 37 °C for 60 min [15, 16]. The remaining total nucleic acid was then isolated using the QIAamp Viral RNA Mini Kit (Qiagen) according to manufacturer's protocol without DNA degradation. For RNA viruses, the RNA was reverse transcribed to cDNA using a reverse transcription kit (SuperScript III Reverse Transcriptase) with oligo dT primers, followed by the addition of a large fragment of DNA polymerase I (Klenow fragment) for the synthesis of second strand cDNA (dsDNA). Specifically, 12 μL nucleic acid extracts were added to the reaction system for synthesizing dsDNA (total reaction system: 20 μL). For ssDNA viruses, the ssDNA was converted to dsDNA by the Klenow reaction and all dsDNA products were used to construct the library. Overall, 36 libraries were constructed using the Nextera XT DNA Sample Preparation Kit (Illumina), and the 300 bp pair-end reads generated by MiSeq were de-barcoded in bioinformatics analysis using Illumina vendor software [17].

### Bioinformatics analysis

An in-house analysis pipeline running on a 32-node Linux cluster was used to process the data. Clone reads were removed and low sequencing quality tails were trimmed using a Phred quality score of 10 as the threshold. Adapters were trimmed using the default parameters of VecScreen (https://www.ncbi.nlm.nih.gov/tools/vecscreen/) which is NCBI BLASTn with specialized parameters designed for adapter removal. The cleaned reads were de novo assembled with each barcode, detected chimera are filtered by length using the ENSEMBLE assembler (https://github.com/xutaodeng/EnsembleAssembler) with the default parameters [18]. Contigs and unassembled reads are then matched against a customized viral proteome database using BLASTx with an E-value cutoff of < 10^−5^, where the virus BLASTx database was compiled using NCBI virus reference proteome (ftp://ftp.ncbi.nih.gov/refseq/release/viral/) to which was added viral proteins sequences from NCBI nr fasta file (based on annotation taxonomy in Virus Kingdom) during data analysis from 2022.1 to 2022.8. Candidate viral hits were then compared to an in-house non-virus non-redundant (NVNR) protein database to remove false positive viral hits, The NVNR database was compiled using non-viral protein sequences extracted from NCBI nr fasta files (based on taxonomy classification, excluding Virus Kingdom). In the vFam database ( http://derisilab.ucsf.edu/software/vFam) [19], HMMER3 v3.2.1 [20, 21] was used to detect remote viral protein similarities.

### Viral sequences extension and annotation

We obtained the contigs by de novo assembly of all cleaned reads using the Low Sensitivity/Fastest parameter in Geneious Prime® 2019.0.4 [22] software, then mapping contigs with the original data using the Low Sensitivity/Fastest parameters in Align/Assemble function in Geneious Prime® 2019.0.4. The open reading frames of the viruses were initially predicted using the Find ORFs function in Genious software (Minimum size: 300; Genetic code: Standard; Start codons: ATG), further the predicted ORFs were compared against the nr database from NCBI using BLASTp. The annotations of these ORFs were based on comparisons to the Conserved Domain Database using RPS-BLAST with an E-value cutoff of < 10^–5^. Contigs annotated with viral marker genes of major virus taxa were selected, and the complete ORFs identified among them were used for further phylogenetic analysis [23]. Nested PCR was performed on the virus belonging to the *Picornaviridae* family without complete ORFs, and specific PCR primers were designed by the Design New Primers program of Genious software based on the available fragment sequences. PCR conditions were as follows: pre-denaturation at 95 °C for 5 min, denaturation at 95 °C for 31 cycles of 30 s, annealing at 50 °C (first round) or 55 °C (second round) for 30 s, extension at 72 °C for 30—110 s (depending on the length of the fragment to be amplified) and a final extension at 72 °C for 5 min. The premixed enzyme rTaq (TaKaRa) was used in the reaction system.

### Phylogenetic analysis

Phylogenetic analysis was based on the predicted amino acid sequences in this study, the closest viral relatives based on BLASTx searches in GenBank, and representative members of related viral species or genera. Related protein sequences were aligned using MUSCLE in MEGA v10.1.8 [24] with the default settings. Sites containing more than 50% gaps were temporarily removed from the alignment. Bayesian inference trees were then constructed using MrBayes v3.2.7 [25]. We set ‘prset aamodelpr = mixed’ for the phylogenetic analysis based on the protein sequences, with two simultaneous runs of Markov chain Monte Carlo (MCMC) sampling in MrBayes. The runs were terminated until the standard deviation of the split frequencies < 0.01, and the first 25% of trees were discarded as burn-in. Maximum Likelihood trees were also constructed to confirm all the Bayesian inference trees using software MEGA v10.1.8 [26] with 1000 bootstrap replicates under the TN93 substitution model and gamma-distributed with invariant sites (G + I). The phylogenetic tree was annotated and embellished using iTOL (https:// iTOL.embl.de/) and Adobe Illustrator.

### Viral community analysis

We used Megan Software (MEtaGenome Analyzer, V6.21.16) to process the results of a comparison of reads against a database of known sequences. The program assigned reads to taxa using the LCA algorithm and then displayed the induced taxonomy. Nodes in the taxonomy can be collapsed or expanded to produce summaries at different levels of the taxonomy. Composition similarity analysis of the seven viromes were compared using Megan Software (MEtaGenome Analyzer, V6.21.16) under the compare option [27]. The Un-weighted Pair Group Method with Arithmetic Mean (UPGMA) taxonomic tree, canonical correspondence analysis (CCA) under cluster analysis option, and Bray–Curtis ecological distance matrix with default parameters were used to present the results. The Friedman rank sum test and SPSS (IBM SPSS 25.0, SPSS Inc) were used to analyze the structural differences among the seven viromes. To visualize the similarity between samples, principal coordinate analysis (PCOA) of R v4.2.2 is used, which is implemented by the function pcoa in the ape package (version 5.3) ( https://CRAN.R-project.org/package=ape), the results of viral community structure and abundance were visualized by heatmap, Venn diagram and histogram, respectively. They were generated by using R v4.2.2 package pheatmap (v1.0.12, https://cran.r-project.org/package¼pheatmap), Venn (v1.9, https://cran.r-project.org/package¼venn) and ggplot2 (v3.2.1, https://ggplot2.tidyverse.org).

### Quality control

To rule out the possibility of nucleic acid contamination in the lab, sterile ddH_2_O (Sangon Biotech) was prepared and processed further under the same conditions as a blank control. Standard precautions were used in all steps to prevent cross-sample contamination and nucleic acid degradation. We used aerosol filter pipet tips to reduce the possibility of sample cross contamination. All materials in direct contact with the nucleic acid samples (including microcentrifuge tubes, pipette tips, etc.) were free of RNase and DNase. Nucleic acid samples were dissolved in DEPC-treated water with RNase inhibitors. All experimental procedures were performed in a biosafety cabinet.

## Results

### Overview of* Virome*

To explore the viral community of herbivorous mammals in the Northwest Plateau, we extracted 36 nucleic acid libraries from seven herbivorous mammalian species, comprising 298 pooled samples in the Northwest Plateau region. After library construction and next-generation sequencing on the Illumina Miseq platform, the 36 libraries yielded a total of 12,299,188 raw reads with an average length of 301 bp and an average GC% of 46.53%. The remaining reads which has been QC analyzed (*N* = 12,109,411) were trimmed within each barcode and assembled from scratch, and the resulting sequence contigs and singlets were then compared to the Viral Reference Database and the GenBank non-redundant protein database using BLASTx with an E-value cut-off of 10^–5^. In total, 33,828 viral contigs were obtained through de novo assembly within the six bins and alignment against the viral protein database using BLASTx, and the average contig length was 551.5 bp (Supplementary Table S1).

### Composition and comparison of viral communities

**Fig.1:**
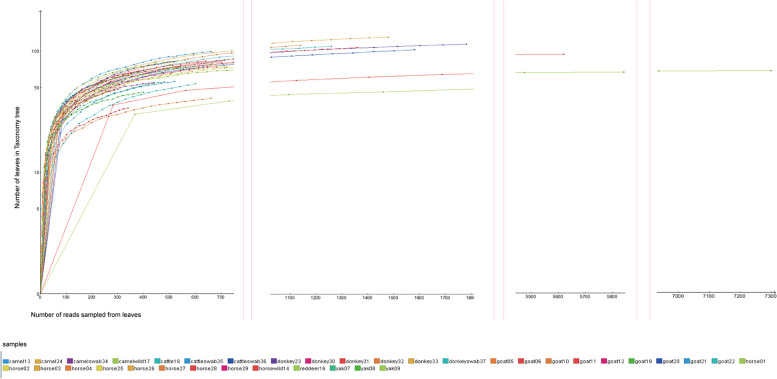
Rarefaction curves of 36 libraries of this study

**Fig.2:**
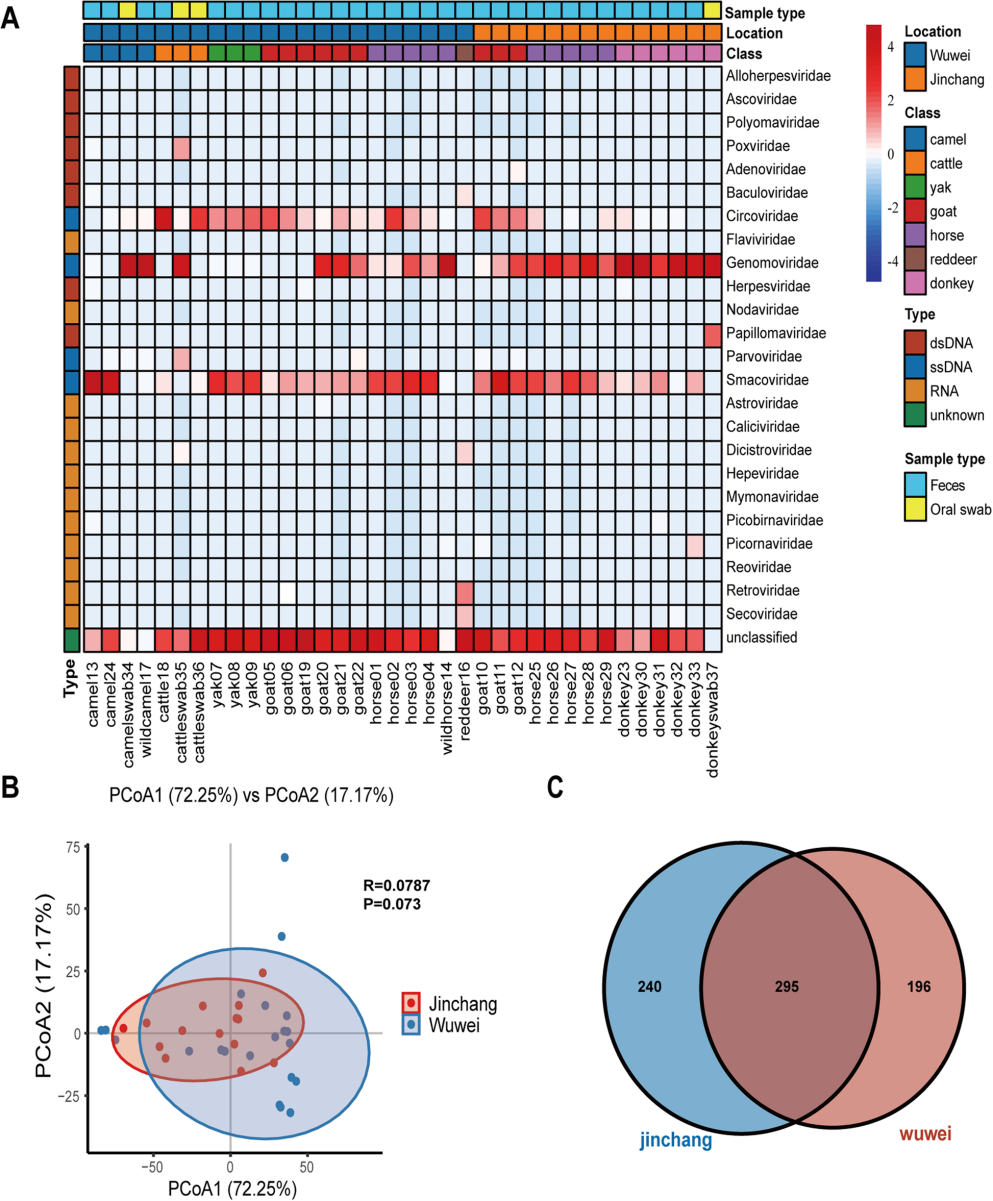
Taxonomic analysis of viral metagenomic reads from the perspective of different animal species. (**A**) Heatmap representing the reads number of each viral family in each herbivorous mammals sample.Sampling location, sample class and viral types are shown with corresponding colors (see color legend). the read abundance of different viral families in each library are shown from blue to red which represent an increasing tendency. (**B**) PCoA plot showing the similarity of viral community structures at each sampling location. (**C**) Venn diagram depicting the distribution of shared and distinct virus sequences among the two locations

Rarefaction curves of the 36 libraries tended to flatten at the end, indicating that the sequencing depth might be sufficient to capture almost all known viral species in the samples, so that the sequencing data are reasonable and convincing (Fig. 1). Contigs and singlets reads with similar viral sequences were grouped into 24 viral families, including 8 dsDNA viral families, 4 ssDNA viral families and 12 RNA viral families. Heatmap at the family level, depending on the sampling area, showed that both Wuwei and Jinchang were dominated by circular replication-associated protein (Rep)-encoding single-stranded (CRESS) DNA viruses including viruses such as genomoviruses, smacoviruses and circoviruses, Besides, *Poxviridae* and *Parvoviridae* were the dominant viral families in Wuwei, and *Papillomaviridae* and *Picornaviridae* were more predominant in Jinchang (Fig. 2A). Plotting the regional principal coordinates analysis (PCoA) analysis at the level of virus families showed that the virus community composition was relatively similar in the two regions, with no statistically significant differences at the family level (*P* = 0.073) (*P* > 0.05) (Fig. 2B). The Venn diagram showed that of the 731 virus sequences detected in the two regions, 295 viruses are common to both regions, accounting for 55.14% and 60.08% in Wuwei and Jinchang respectively (Fig. 2C).

**Fig.3:**
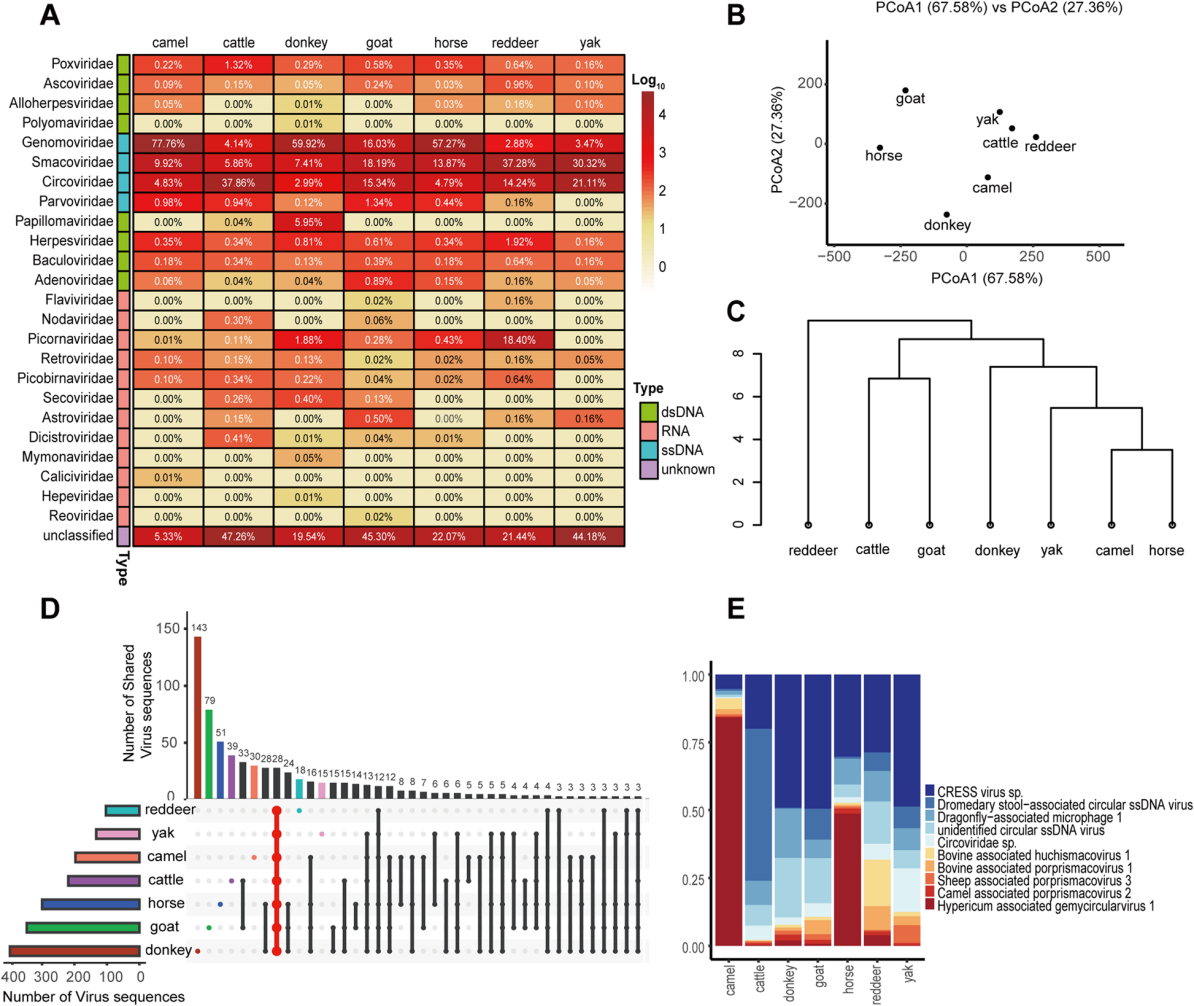
Taxonomic analysis of viral metagenomic reads from an animal perspective. (**A**) Heatmap representing the reads number of each viral family in each herbivorous mammals sample.Viral types are shown with corresponding colors (see color legend).The percentage of each vial family in each of the 7 animal species was shown in the corresponding rectangle. (**B**) PCoA plot and (**C**) UPGMA taxonomic tree showing the similarity of viral community structures of 7 animal species. (**D**) UpSet plot depicting the numbers of shared virus sequences among the7 animal species. Filled dots with interconnecting vertical lines represent the intersections, and unfilled light gray dots represent sets that do not belong to the intersections. The bars above represent the numbers of species within the intersections, and the bars to the left depict the total number of virus sequences in each virome set. (**E**) The bar graph showing the top 10 most abundant virus sequences in the seven viromes

Depending on the animal species, the 36 libraries were divided into 7 groups to plot the heatmap (Fig. 3A). Differences in virus abundance levels could be seen between the seven animal species. the greatest abundance was found in horse which was 30.61%, while red deer had the lowest abundance which was 1.54%. For the reads of different virus families in each animal species, *Genomoviridae* was the dominant family in camel, donkey, and horse, most virus reads were assigned to *Smacoviridae* family in goat, red deer, and yak, and viruses belonging to the family *Circoviridae* accounted for the largest proportion of cattle. Papillomaviruses and picornaviruses also accounted for a certain proportion of donkey and red deer, respectively, and were the more distinctive viral families of the two animal species. Overall, the majority of viral reads from the seven animal species were ssDNA and were dominated by viral families homologous to CRESS-DNA viruses, a smaller proportion of sequences belonging to the *Parvoviridae* family were also present, and a comparison of virus sequences assigning to the *Parvoviridae* family from seven animal species revealed that there were more parvoviruses in goat. Relatively few sequences classified into dsDNA and RNA. The most dominant viral family in dsDNA was *Poxviridae*. Comparative analysis were performed on the viral families in seven animal species to evaluate uniqueness and convergence between them. Principal Coordinates Analysis (PCoA) analysis (Fig. 3B) and UPGMA dendrogram (Fig. 3C) showed a clear separation between red deer and the other six animal species.

The Upsetplot (Fig. 3D) showed that a total of 749 virus sequences were detected in the seven animal species, with each animal species having a certain number of unique viruses, donkey had the highest number of unique viruses with 143 virus sequences, while yak had the lowest number with 15 virus sequences. At the same time, 28 virus sequences were shared by the seven animal species, accounting for 21.63% to 75.22% of the total number of viruses in each of the seven animal species, mostly above 50%. It indicated that the viral communities of the seven animal species were relatively similar. For further study, the results of the top 10 virus sequences shared by the seven animal species were shown (Fig. 3E), with more than half of the virus sequences being CRESS-DNA viruses. It appears that CRESS-DNA viruses are the dominant viruses in herbivorous mammals of the Northwest Plateau and that CRESS-DNA viruses have a large species diversity.

### Identification of virus hallmark gene sequences

By sequence extension and annotation, a total of 81 viral sequences with complete CDS were obtained in this study, and all sequences were classified into five groups based on reads abundance and conserved structural domains (i.e. viral signature genes), namely CRESS-DNA viruses of replication protein (Rep), major capsid protein (MCP) of *Microviridae*, Polyprotein and the P1, P3 functional regions of *Picornaviridae*, the RNA dependent RNA polymerase protein (RdRp) structural domain of *Nodaviridae* and the capsid protein (CP) functional region of *Pepper mild mottle virus* (PMMoV), the identity of 24 CRESS-DNA viruses with their best matched known viruses were from 37.71% to 95.68%, with 14 out of 24 CRESS-DNA viruses below 75% identity. At the same time, only 12 out of 51 microviruses had a similarity of over 50% with the best matched known microviruses. Two picornaviruses found in this study showed 93.32% and 52.99% identity with known viruses, and the identity of nodavirus and pepper mild mottle viruses with their best matched viruses was nearly 100%.

### Phylogenetic analyses of viral sequences

**Fig.4:**
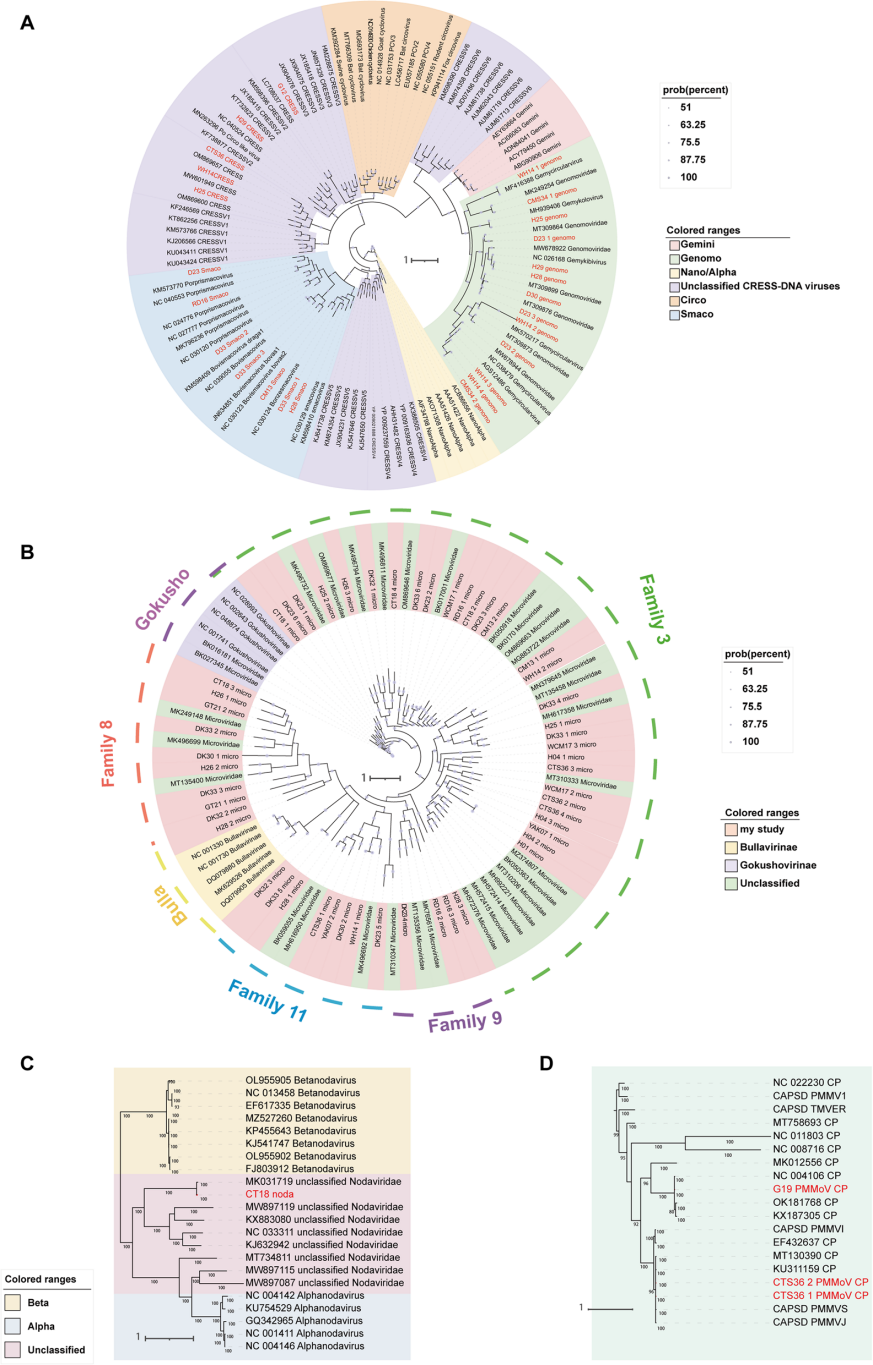
Phylogenetic analysis of CRESS-DNA viruses, *Microviridae*, *Nodaviridae* and *Pepper mild mottle virus* (PMMoV) sequences. (**A**) Bayesian inference tree established based on amino acid sequences of Rep protein of CRESS-DNA viruses. (**B**) Bayesian inference tree established based on amino acid sequences of MCP of *Microviridae*. (**C**) Bayesian inference tree established based on amino acid sequences of RdRp protein of *Microviridae*. (**D**) Bayesian inference tree established based on amino acid sequences of CP of PMMoV. Within trees in A,B,C,D,the viruses found in this study are marked with red lines and letters.Each scale bar indicates the amino acid substitutions per site. Different taxonomic clusters were represented by rectangles filled with different colors and taxon names are indicated on the left with the corresponding colors (see color legend). (**A**) and (**B**) bootstrap values > 50 are indicated on the trees and the size of dots on nodes correspond to the bootstrap values

In this study, a total of 81 sequences with complete viral marker genes and one sequence with the nearly complete marker region were screened out, and phylogenetic analysis were performed based on the amino acid sequences encoded by the viral marker genes and the corresponding reference protein sequences. ICTV criteria for new genus or species of viruses identified in this study was provided in Supplementary Table 3. CRESS-DNA viruses are widely distributed in many ecosystems, geminiviruses and nanoviruses infect plants, while circoviruses infect both vertebrates (mammals and birds) and invertebrates. We also found 24 CRESS-DNA viruses with the complete Rep protein sequence in seven herbivorous mammals of this study. A phylogenetic tree of CRESS-DNA viruses (Fig. 4A) was drawn based on 24 replication protein (Rep) sequences, revealing an undetected species diversity of CRESS-DNA viruses, five of which belonged to unclassified CRESS-DNA viruses, seven of which were phylogenetically clustered with known viral strains in the *Smacoviridae* branch with 43.54%-76.26% Rep protein sequence identity, and the remaining sequences clustered with *Genomoviridae* with 70.67%-95.88% sequence identity. These sequences contain sequences from five animal species in this study: sheep, horse, cattle, donkey and red deer, further demonstrating that the host range of CRESS-DNA viruses is large. Considering that CRESS-DNA viruses are found in the viral groups of many ecosystems and are known to infect a wide range of organisms like plant, bird and mammals, these CRESS-DNA viruses may also from the diet-contaminants.

Microviruses represent the largest class of ssDNA phages, and this study identified 51 sequences with complete viral marker genes, they encompass all animal species in this study. A phylogenetic tree (Fig. 4B) based on the 51 MCPs of the *Microviridae* revealed a large amount of genetic diversity in ssDNA phages. Specifically, all sequences did not establish clear associations with the subfamilies *Bullavirinae* and *Gokushovirinae* and were genetically divided into different branches from the two subfamilies. This result reveals a diversity of unidentified phages in herbivorous mammals of the Northwest Plateau.

In addition, two RNA viruses, viruses belonging to the *Nodaviridae* family and *Pepper mild mottle virus* (PMMoV), were also found in the virus community of herbivorous mammals in the Northwest Plateau. A phylogenetic tree was constructed based on the RdRp structural domain of *Nodaviridae* (Fig. 4C) and the CP protein sequence of *Pepper mild mottle virus* (PMMoV) (Fig. 4D). The nodavirus found in cattle belonged to the unclassified *Nodaviridae*, which was 97.19% similar to the *Nodaviridae* found in bovine lung (GenBank No. MK031719). A total of three complete *Pepper mild mottle virus* (PMMoV) sequences were obtained from goat and cattle, and Blastx in GenBank nr DB revealed close to 100% identity with known *Pepper mild mottle virus* (PMMoV).

**Fig.5:**
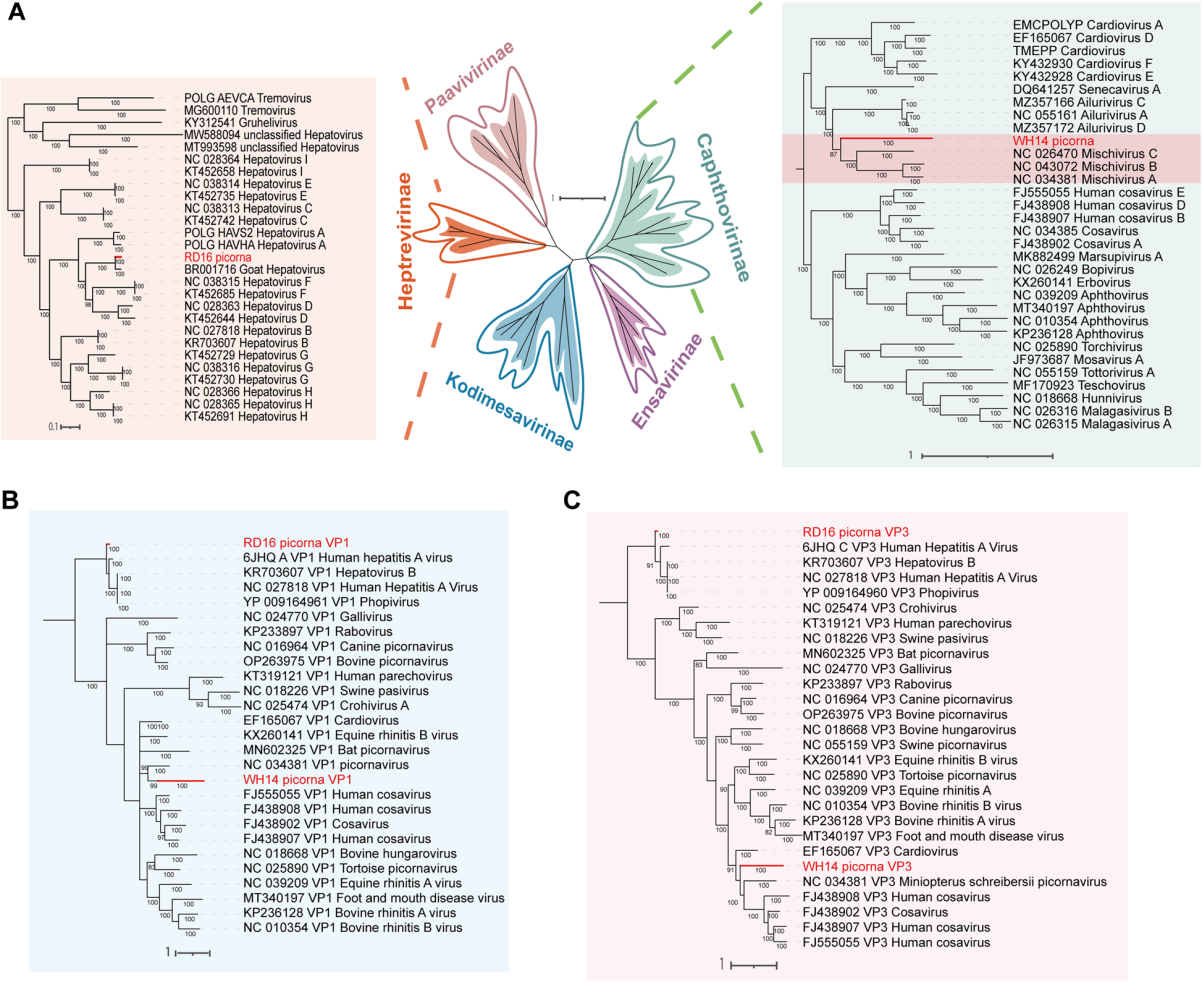
Phylogenetic analysis of picornaviruses sequences. (**A**) Bayesian inference tree established based on amino acid sequences of polyprotein of *Picornaviridae*. (**B**) Bayesian inference tree established based on amino acid sequences of VP1 of *Picornaviridae*. (**C**) Bayesian inference tree established based on amino acid sequences of VP3 of *Picornaviridae*.Within trees in A,B,C, the viruses found in this study are marked with red lines and letters.Each scale bar indicates the amino acid substitutions per site

Two picornaviruses were identified in wild horse and red deer, and phylogenetic trees (Fig. 5) were constructed based on the protein sequences of the polyprotein, P1, and P3 functional regions. The picornavirus detected in red deer was found to be closely clustered with the *Hepatovirus* species which was belonged to the *Heptrevirinae* genus and showed 93.32% similarity at the nucleotide level to the Goat hepatovirus (GenBank No. BR001716), which, as far as we know, is the first time that a hepatovirus has been identified in red deer, suggesting that hepatovirus might be able to transmit across animal species. However, the wild horse picornavirus is phylogenetically classified as *Caphthovirinae* and is closely related to *Mischivirus*, with the closest virus being Miniopterus schreibersii picornavirus 1 (GenBank No. NC_034381), a picornavirus found in bats [28], with a coverage of 75% and an identity of only 52.99% at the nucleotide level, it is reasonable to suspect that the picornavirus found in wild horse is a novel picornavirus that have not been found before.

## Discussion

Herbivorous mammals in the Northwest Plateau carry a wide variety of viruses, and one study found that most human diseases originate in animals (61%), of which 70% are emerging [29]. Given the large number of human populations and the high abundance of herbivorous mammals, the expansion of animal-human interface and the fragile ecological environment, viruses carried by herbivorous mammals on the Northwest Plateau are likely to infect humans and cause human infectious diseases. In this study, we collected a total of 298 fecal and oral swab samples from seven herbivorous mammals from multiple sites in Wuwei and Jinchang, Gansu Province, as representative areas of the Northwest Plateau, to gain a better understanding of the viral communities in herbivorous mammals and the potential impact on human life and health.

The results showed that the composition of herbivorous mammalian virus communities in the Northwest Plateau was largely similar regionally, but there were slight differences between 7 animal species. The overall virus communities in the two regions of Wuwei and Jinchang were relatively similar, both being dominated by CRESS-DNA viruses, which are circular single-stranded DNA viruses encoding replication-associated proteins including viruses such as genomoviruses, smacoviruses and circoviruses. CRESS-DNA viruses are found in the viral groups of many ecosystems and are known to infect a wide range of organisms like plant, bird and mammals, geminiviruses and nanoviruses infect plants, while circoviruses infect both vertebrates (mammals and birds) and invertebrates [30]. It is therefore not surprising that CRESS-DNA viruses are the dominant viruses in both regions.

It is also necessary to compare virus communities in different herbivorous mammals. In terms of viral taxa diversity, the virus communities of all seven herbivorous mammals were dominated by ssDNA. CRESS-DNA viruses accounted for the largest proportion. CRESS-DNA viruses have now been found to be associated with some cases of human diarrhoea [31], and may infect food ingested by humans, such as consumption of virus-infected pork. Several studies have identified that Porcine circovirus 2(PCV2) is a major pathogen in a series of syndromes called Porcine circovirus type 2 (PCV2)–associated disease (PCVAD), including respiratory disease, intestinal disease (porcine dermatitis and nephrotic syndrome) and reproductive problems [32]. Other studies have isolated CRESS-DNA viruses in the cerebrospinal fluid of patients with acute central nervous system infections [33]. Although no studies have definitively shown that CRESS-DNA viruses cause diseases in humans, based on previous studies and the genetic diversity of the CRESS-DNA viruses, CRESS-DNA viruses are associated with the healthy life of human beings. In addition to CRESS-DNA viruses, viruses belonging to the *Parvoviridae* family were also identified by bioinformatics analysis, and goat contained the most parvoviruses among the seven animal species. Parvoviruses are genetically diverse non-enveloped viruses that can infect a wide range of animals from insects to humans. A study has identified parvovirus genomes closely related to the human fecal-associated parvovirus gene in fecal samples from Hungarian goats and sheep, with > 97% and 95–100% amino acid sequence identity in the parvoviral NS1 and VP1 protein, tusaviruses (OL692339-OL692348) from goat and sheep are very closely related to each other and to the human tusavirus (KJ495710) based on phylogenetic analysis of VP1 aa sequences [34]. Thus, the parvovirus infection in humans may be zoonotic. Although we identified parvoviruses, we did not obtain a more complete genome sequence to determine exactly what virus they were, possibly due to the low concentration of parvoviruses contained in the samples collected, resulting in an insufficient number of reads assembled into longer contigs for phylogenetic analysis. It is noteworthy that red deer contains particularly high levels of picornaviruses compared to other animals. Picornaviruses are important human and animal pathogens that cause diseases affecting the central nervous system, respiratory and gastrointestinal tracts [35], heart [36], liver [37], and are closely associated with acute infectious diseases such as Poliomyelitis [38] and foot-and-mouth disease [39]. The UPGMA dendrogram clearly distinguished red deer from the other six animal species, suggesting that red deer had a unique viral community. A total of 749 virus sequences were detected in the seven herbivorous mammals, with each animal species having a certain number of unique virus sequences. The Upsetplot (Fig. 3D) showed that donkey had the highest number of unique viruses with 143 virus sequences, while yak had the lowest number with 15 virus sequences, demonstrating that the virus communities of herbivorous mammals are somewhat different from each other, reflecting the diversity of viruses carried by herbivorous mammals.

Viral marker genes are genes that are relatively conserved in a given viral group [40]. In this study, phylogenetic analyses based on the amino acid sequences encoded by viral marker genes and corresponding reference protein sequences were performed to reveal the diversity of viruses carried by herbivorous mammals in the Northwest Plateau. Twenty-four CRESS-DNA viruses with complete Rep functional regions were obtained from five animal species, most of which clustered closely with known viral species of the *Genomoviridae* and *Smacoviridae* family, but two sequences remained clustered with unclassified CRESS-DNA viruses, and three sequences were located between known clusters, forming new clusters, reflecting the genetic diversity of CRESS-DNA viruses, there are still many unknown CRESS-DNA viruses in herbivorous mammals waiting to be discovered. Microviruses are representative ss-DNA phages. Microviral diversity can be partitioned into 19 families that encompass over 99% of known microviral diversity, and the 19 families can be assorted into three suborders. Suborder I includes Family 1 and Suborder II encompasses Family 2, while Family 3 to Family 19 belong to Suborder III [41]. Fifty-one sequences of microviruses were obtained in this study after sequence assembly and amplification. The phylogenetic analysis revealed that all microvirus sequences were clustered with Suborder III, with 29 microvirus sequences belonging to Family 3, 10 sequences classified into Family 8, 4 sequences clustered with Family 9 and 10 sequences belonging to Family 11. This indicates that the diversity and quantity of microviruses are unimaginable, which is worth further exploration.

In addition to DNA viruses, nodaviruses, pepper mild mottle viruses and picornaviruses were RNA viruses that we performed by phylogenetic analysis. The host range of nodaviruses is insects (*Alphanodavirus*) or fish (*Betanodavirus*) [42], and no studies have demonstrated that nodaviruses are capable of infecting herbivorous mammals. The CRESS-DNA viruses and nodaviruses are believed to infect plants and insects, and microviruses can infect bacteria, identifying that they were likely from the diet of herbivorous mammals. *Pepper mild mottle virus* (PMMoV) is a non-enveloped, rod-shaped, single-stranded positive sense RNA virus belonging to the genus *Tobamovirus*. Tobamoviruses are highly concentrated and very stable in infected plants [43]. It has been suggested that *Pepper mild mottle virus* (PMMoV) may not only be a common inhabitant of the human gut, but may also interact with the human immune system and cause clinical symptoms such as fever, abdominal pain and pruritus [44]. Similarly, the detection of *Pepper mild mottle virus* (PMMoV) in herbivorous mammals in the Northwest Plateau in this study suggests that *Pepper mild mottle virus* (PMMoV) is also present in herbivorous mammals and may have an impact on their health.

Picornaviruses contain many pathogenic viruses, and two picornaviruses were identified from red deer and wild horse. The picornavirus detected in red deer clustered with the subfamily *Heptrevirinae*, the closest being hepatitis A virus (HAV) found in goat samples [45]. We BLAST the capsid protein and RNA dependent RNA polymerase protein (RdRp) of red deer HAV and human HAV (GenBank No. A3FMB2), finding that the similarity of deer hepatitis A virus capsid protein and human hepatitis A virus capsid protein was 84%, and the similarity of RdRp functional regions between two hepatitis A viruses was 62%, which reflected that the red deer hepatitis A virus had the relatively high similarity with human hepatitis A virus. This virus is likely to pose a threat to the health of herbivorous mammals in the Northwest Plateau. A number of pathogenic viruses have been identified from red deer. Red deer is an important host of ticks and a major vector of Crimean-Congo haemorrhagic fever virus (CCHFV) [46] and tick-borne encephalitis virus (TBEV) [47]. The role of red deer in viruses such as hepatitis E virus (HEV) [48], foot-and-mouth disease virus (FMDV) [49] and bluetongue virus (BTV) [50] has also received considerable attention. However, according to our knowledge, no studies have found hepatitis A virus (HAV) in red deer, and this finding adds to the knowledge of viruses carried by red deer and broadens the understanding of the genetic diversity of hepatitis A virus (HAV). The picornavirus carried by wild horse was phylogenetically assigned to the bat-related Mischivirus, with 52.99%, 49.65% and 43.31% similarity to Mischivirus A (GenBank No. NC_034381), Mischivirus B (GenBank No. MG888045) and Mischivirus C (GenBank No. NC_026470), respectively, suggesting that this could potentially form a new species within the *Picornaviridae* family [51]. The result of the alignments were shown in Supplementary Table 2. Interestingly, samples from both red deer and wild horse were collected from the same safari park, so it is likely that there is an aggregation of picornaviruses infections in this safari park, having a potential risk of cross-species transmission of picornaviruses, and wild mammals play an important role [52] in zoonotic diseases.

## Conclusion

In conclusion, this study systematically investigated the viral communities of herbivorous mammals in the Northwest Plateau and potential viruses in herbivorous mammals that could affect human health were initially explored. The viral communities in Wuwei and Jinchang were not very different, but there were differences in viruses between animal species. CRESS-DNA viruses, parvoviruses, and picornaviruses are the viruses identified in this study that potentially affect human health. There are some limitations of my study, it is descriptive and most of our genomes are partial thus cannot be robustly classified. Although the relationship between these potentially pathogenic viruses and human health could not be clarified due to limitations in sample coverage and sequencing, this study greatly expands the knowledge of virus communities in various herbivorous mammals and encourages future studies to focus on potentially pathogenic virus groups in herbivorous mammals of the Northwest Plateau.

### Supplementary Information

**Additional file 1: Supplementary Figure 1. **PCoA plot of goat and horse of two regions.

**Additional file 2: Supplementary Figure 2. **The accumulation curve of 36 libraries of this study.

**Additional file 3: Supplementary Figure 3. **Viral reads of each family of three negative controls. The abundance was shown as the actual number of viral reads in each negative control library.

**Additional file 4: Supplementary Table 1. **Information of sampling sites and corresponding libraries.

**Additional file 5: Supplementary Table 2. **Information of viral sequences with virus hallmark genes identified in herbivorous mammalian.

**Additional file 6: Supplementary Table 3. **ICTV criteria for new genus or species of viruses identified in this study.

**Additional file 7: Supplementary Table 4. **Information of negative controls.

## Data Availability

The raw sequence reads data analyzed in this study are available at the National Center for Biotechnology Information (NCBI) Sequence Read Archive database under the accession numbers SRR22799473 to SRR22799508 (Supplementary Table S1). All viral sequences with virus hallmark genes identified in this study were deposited in the GenBank database under the accession numbers listed in Supplementary Table S2.
