# Supplementary material for: Metagenomic analysis of herbivorous mammalian viral communities in the Northwest Plateau
Source: BMC Genomics. 2023 Sep 25;24:568. doi: 10.1186/s12864-023-09646-1 (PMC10521573; doi:10.1186/s12864-023-09646-1)
Supplement: Supplementary file 1 — Additional file 1: Supplementary Figure 1. PCoA plot of goat and horse of two regions. [file 12864_2023_9646_MOESM1_ESM.pdf]

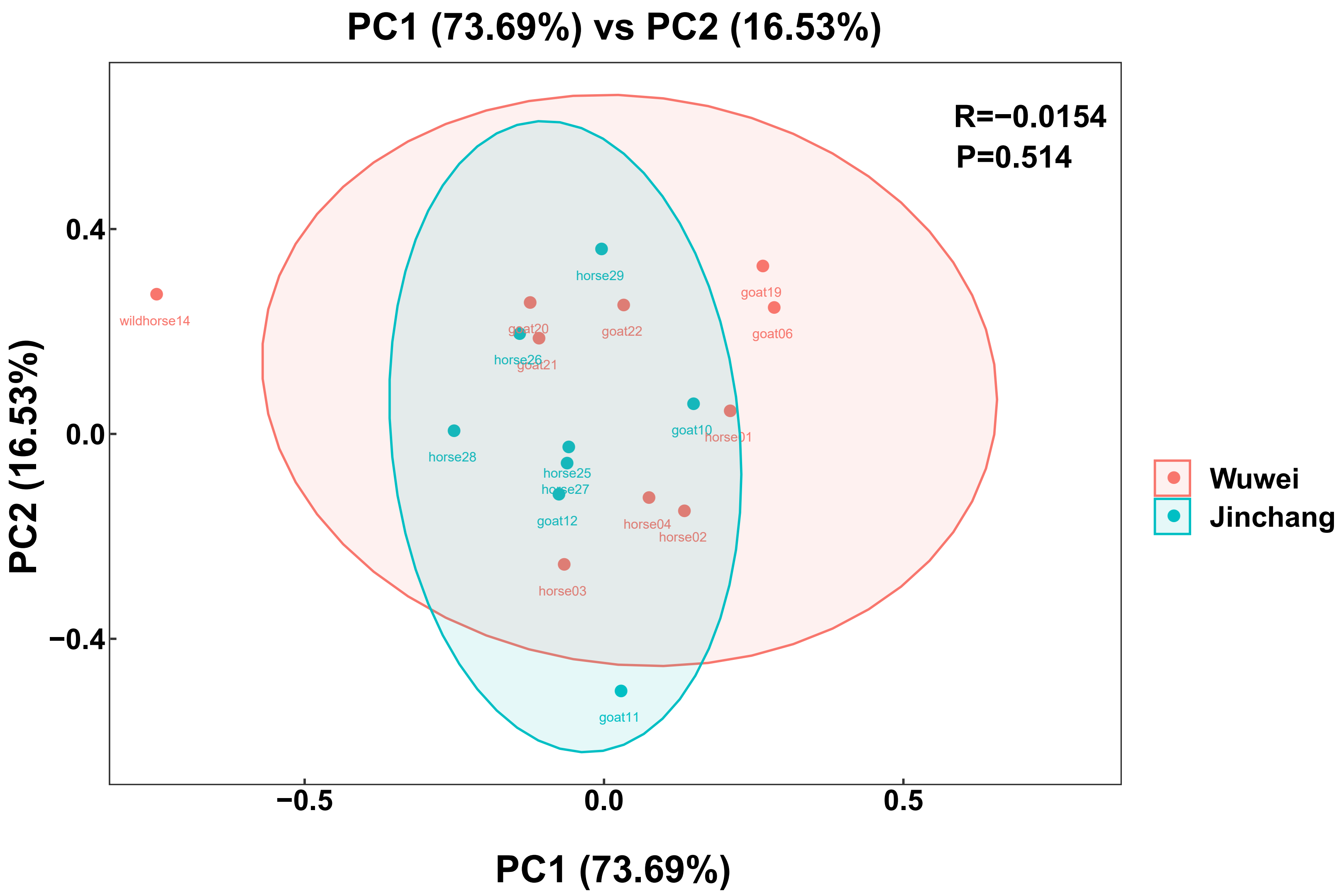

Supplementary Figure 1. PCoA plot of goat and horse of two regions.
PCoA plot showing the similarity of viral community structures of goat and horse at each sampling location.
