# Supplementary figures and images for: Metagenomic analysis of herbivorous mammalian viral communities in the Northwest Plateau
Source: BMC Genomics. 2023 Sep 25;24:568. doi: 10.1186/s12864-023-09646-1 (PMC10521573; doi:10.1186/s12864-023-09646-1)

## Accumulation curve

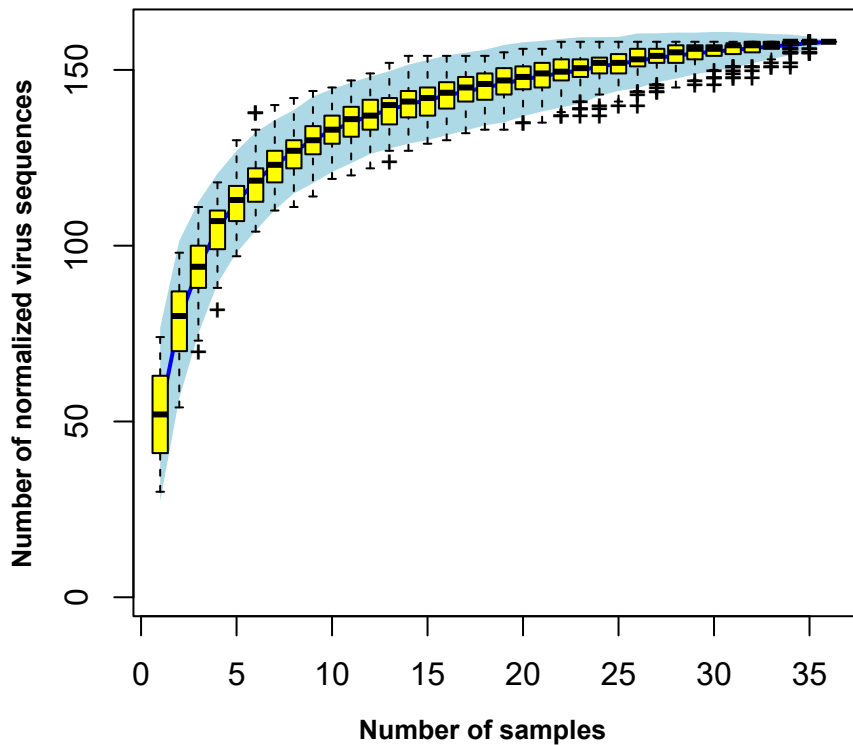

**Supplementary Figure 2.** The accumulation curve of 36 libraries of this study

Supplement: Supplementary file 2 — Additional file 2: Supplementary Figure 2. The accumulation curve of 36 libraries of this study. [file 12864_2023_9646_MOESM2_ESM.pdf]
