# Supplementary material for: Metagenomic analysis of herbivorous mammalian viral communities in the Northwest Plateau
Source: BMC Genomics. 2023 Sep 25;24:568. doi: 10.1186/s12864-023-09646-1 (PMC10521573; doi:10.1186/s12864-023-09646-1)
Supplement: Supplementary file 3 — Additional file 3: Supplementary Figure 3. Viral reads of each family of three negative controls. The abundance was shown as the actual number of viral reads in each negative control library. [file 12864_2023_9646_MOESM3_ESM.pdf]

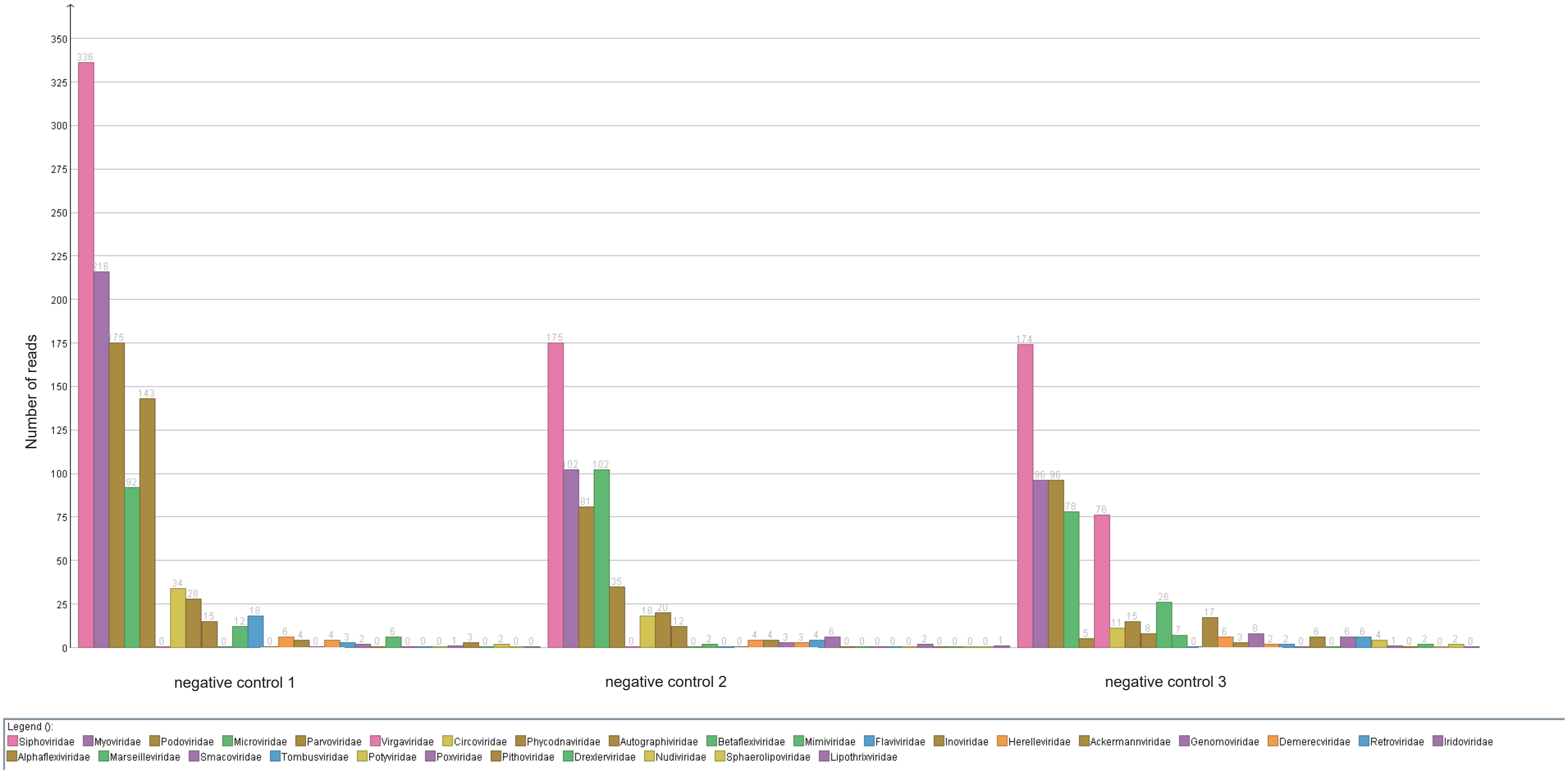

Supplementary Figure 3. Viral reads of each family of three negative controls. The abundance was shown as the actual number of viral reads in each negative control library.
